# The Relationship between Risk Factors for Metabolic Syndrome and Bone Mineral Density in Menopausal Korean Women

**Published:** 2019-06

**Authors:** Seok-Hee KIM, Jooyoung KIM

**Affiliations:** 1. School of Humanities and Social Science, College of Liberal Arts and Convergence Sciences, Korea Advanced Institute of Science and Technology, Daejeon-si, Republic of Korea; 2. Department of Anatomy, School of Medicine, Kyungpook National University, Daegu-si, Korea

**Keywords:** Bone mineral density, Menopausal women, Metabolic syndrome, Osteoporosis, Korea

## Abstract

**Background:**

The risk factors of metabolic syndrome (MetS) in menopausal women are potential causes of osteoporosis. However, there is no consensus on this. We aimed to determine the relationship between risk factors of MetS and bone mineral density (BMD) in menopausal Korean women.

**Methods:**

We enrolled 205 menopausal Korean women who visited a health promotion center in Seoul in 2015 and divided them into the following two groups according to the National Cholesterol Education Program-Adult Treatment Panel III (NCEP-ATP III) criteria with modified waist-circumference criteria: the non-MetS group (Group 1, n=90) and the MetS group (Group 2, n=115). Anthropometric parameters and clinical parameters, including blood pressure, blood lipid profile (cholesterol, triglycerides), and fasting blood sugar levels were recorded for all participants. BMD at the lumbar spine was determined using dual-energy X-ray absorptiometry (DEXA). The relationship between the risk factors of MetS and bone mineral density was analyzed by statistical methods.

**Results:**

There was no significant difference in risk factors of MetS between the groups. In correlation tests, waist circumference showed a significant association with body surface area (BSA) (r = −0.242, *P* < 0.001). Diastolic blood pressure was correlated with BSA (r = 0.186, *P* < 0.01) and bone mineral content (BMC) (r = 0.161, *P* < 0.05). However, multiple regression analysis showed no significant relationship between MetS risk factors and BMD.

**Conclusion:**

The risk factors of MetS did not affect BMD in menopausal Korean women. Follow-up studies with a larger study population are necessary size to allow the investigation of other research variables.

## Introduction

Metabolic syndrome (MetS) is a disorder characterized by abdominal obesity, hyperglycemia, hypertension, and dyslipidemia. It is known to be significantly associated with increased risk of cardiovascular disease and resulting death (1). MetS increases with age, and its prevalence is especially high in menopausal women (2). Estrogen deficiency in menopausal women causes abdominal adipose-tissue accumulation and inflammation, decreases in vascular elasticity and endothelial cell function and increased low-density lipoprotein cholesterol (LDL-C) (3).

Another health problem that may appear in menopausal women is osteoporosis (4). The primary cause of osteoporosis in menopausal women is estrogen deficiency (5). The decrease in estrogen secretion impairs the normal bone turnover cycle, increasing osteoclastic resorption activity while reducing osteoblastic activity (6). As a result, bone loss increases, and the risk of osteoporosis increases. This change is a major cause of reduced quality of life due to increased susceptibility to fractures and is a major threat to health in menopausal women (7).

MetS in menopausal women may promote bone mineral density (BMD) reduction (8–10). The reduction of high-density lipoprotein cholesterol (HDL-C) suppresses osteogenic activity in vascular cells, and abdominal obesity increases inflammatory cytokines such as interleukin-6 (IL-6) and tumor necrosis factor alpha (TNF-α) (10). Increased blood pressure damages calcium homeostasis, partly due to urinary calcium excretion (11). Increased triglycerides reduce hypercalciurina and insulin-like growth factor-1 (IGF-1) and blood flow to the bone, leading to decreased BMD (10, 12).

However, there was no correlation between MetS and decreased BMD in menopausal women (13–16). However, menopausal women with MetS had a higher T-scores of the femoral neck than those without MetS (17), and BMD was higher in menopausal women with lower HDL-C levels (18). In addition, obesity and overweight have been separately reported to have a protective effect on bone loss and thereby reduce the fracture risk (19–21).

Thus, research has shown inconsistent results regarding the relationship between MetS and BMD in menopausal women, and the correlation and causation are still unclear. Nevertheless, considering a report of low BMD in Asian women compared to Caucasians (22) and recent studies (10, 23) that continue to highlight the health problems of menopausal women, especially a decrease in BMD by MetS, it is necessary to make continuous attempts to elucidate the relationship between the two variables. Moreover, while Korea has focused on treatment for menopausal women with osteoporosis, its efforts at prevention by identifying and reducing potential risk factors have been relatively limited (24–25).

Therefore, we aimed to investigate the relationship between BMD and the risk factors of MetS in menopausal Korean women and to provide basic data on BMD decrease and its prevention.

## Materials and Methods

### Subjects

Subjects were 205 menopausal women who visited a health promotion center in Seoul in 2015 and received an examination. Menopause was defined as cessation of menstruation for at least one year. The researchers fully explained the purpose and contents of the study to all study subjects. Then, the subjects voluntarily agreed to participate in the study and signed an informed consent form. Those who were receiving medication or hormone replacement therapy for osteoporosis treatment or had a history of thyroid dysfunction or rheumatoid arthritis were excluded from this study.

The study subjects were divided by diagnosis into a non-MetS group (Group 1, n = 90) and a MetS group (Group 2, n = 115). MetS was diagnosed when three or more risk factors, including blood pressure, blood glucose, triglycerides, HDL-C, and abdominal obesity met specific thresholds. Except for the waist-circumference criterion used to diagnose abdominal obesity, other risk factors of MetS were based on the criteria presented in the National Cholesterol Education Program Adult Treatment Panel III (NCEP-ATP III) (26). The criteria presented by the NCEP-ATP III are as follows: 1) systolic blood pressure (SBP) ≥130 mmHg or diastolic blood pressure (DBP) ≥85 mmHg; 2) fasting blood sugar ≥100 mg/dl; 3) triglycerides ≥150 mg/dl; and 4) high density lipoprotein cholesterol (HDL-C) ≤50 mg/dl. The waist-circumference criterion was modified to take into account the sex and race of the subjects, and was set to be 80 cm or more as suggested by the World Health Organization, West Pacific Region, in 2000 (27). The characteristics of the study subjects in each group are shown in Table 1.

**Table 1:** Differences in BMD-related factors according to the diagnosis of the metabolic syndrome

***Item***	***Group 1 (n=90)***	***Group 2 (n=115)***	***t***
Age (yr)	63.19±3.27	64.36±3.34	−2.502*
Height (cm)	153.91±4.69	153.96±4.95	−.079
Body weight (kg)	56.55±7.14	59.19±6.44	−2.771**
BMI (kg/m^2^)	23.86±2.69	24.97±2.54	−3.019**
WC (cm)	81.62±0.40	83.23±0.38	−2.892**
SBP (mmHg)	119.73±9.75	136.80±14.78	−9.926***
DBP (mmHg)	72.04±8.05	81.43±10.00	−7.253***
TG (mg/dl)	93.34±28.39	155.53±71.68	−7.764***
HDL-C (mg/dl)	62.42±9.38	61.79±10.43	0.449
FBS (mg/dl)	84.53±16.58	89.33±17.03	−2.027*

Values are Mean±standard deviation, Group 1: non-metabolic-syndrome group, Group 2: metabolic-syndrome group, t; result of independent t-test, BMI; body mass index, WC; waist circumference, SBP; systolic blood pressure, DBP; diastolic blood pressure, TG; triglyceride, HDL-C; high density lipoprotein cholesterol, FBS; fasting blood sugar.

* *P<*0.05*,*

** *P<*0.01*,*

*** *P<.*001

### Data collection

Height and weight were measured using an automatic anthropometer (GL-150, G-Tech International Co., Ltd., Uijeongbu, Korea), and the body mass index was calculated as body weight (kg)/height (m^2^). The waist circumference was measured at the midpoint between the lowest rib and the upper iliac crest using a tape measure on exhalation. Blood pressure was measured using an automatic blood pressure monitor (EASY X800 R/L, Jawon Medical Co., Ltd., Gyeongsan, Korea) after resting for 5 minutes in a sitting position before measurement, and the mean value was recorded after two measurements at a 3-min interval. To obtain blood samples for the measurement of fasting blood sugar, high density lipoprotein cholesterol, and triglycerides, blood was collected from a forearm vein between 8 am and 10 am, after an 8-hour fast. All blood samples were analyzed using an Auto Biochemistry Analyzer (Express 550 Plus, Bayer, New Jersey, USA). A dual-energy X-ray absorptiometry (DEXA) unit (Discovery, Hologic Inc, CA, USA) was used for BMD measurements. BMD was measured at the lumbar spine (L1–L4) in a supine position. Total bone mineral content (BMC) values for each area from L1–L4 were presented as grams of bone per cm^2^, and the T-score (comparison between subjects and young normal adults) and Z-score (comparison with subject’s age/sex group) were calculated. The difference between the subject and the normal group was divided by the standard deviation. The T-score was thus calculated as (subject BMD − mean young BMD)/1SD. The value was defined as: normal when > −1.00; osteopenia, −1.00 to −2.49; and, osteoporosis, ≤ −2.50, according to the World Health Organization criteria (28). The Z-score formula was (subject BMD − mean BMD for same age group)/1SD, and calculated values of less than −2.00 required additional examination to investigate a diagnosis of metabolic bone disease (29).

### Statistical analysis

All data in this study are presented as mean (standard deviation). Independent *t*-test was performed to compare the mean difference between the two groups according to the diagnosis of MetS. Pearson correlation was used to analyze the relationship between risk factors of MetS and BMD. Multiple regression analysis was performed to examine the effect of risk factors of MetS on BMD. All data were analyzed using statistical analysis software (SPSS ver. 18.0, IBM Corp., Armonk, NY, USA). Statistical significance was set at *P*<0.05.

## Results

Table 2 shows the differences in BMD-related factors between the groups. There was no significant difference in BMD-related factors between the MetS and non-MetS groups. The levels of T-scores in this study reflected osteopenia, rather than osteoporosis. Table 3 shows the results of the correlation between risk factors of MetS and BMD. In terms of the relationship between risk factors of MetS and BMD factors, a significantly negative correlation was observed between body surface area (BSA) and waist circumference (r= −0.242, *P*<0.001). Diastolic blood pressure showed a positive correlation with BSA (r=0.186, *P*<0.01) and BMC (r=0.161, *P*<0.05).

**Table 2:** Differences in BMD signs grouped by diagnosis of metabolic syndrome

***Variable***	***Group 1 (n=90)***	***Group 2 (n=115)***	***t***
BSA (cm^2^)	53.69±4.45	53.98±4.59	−0.445
BMC (g)	43.78±8.55	45.11±9.71	−1.041
BMD (g/m^2^)	0.81±0.12	0.82±0.13	−1.004
T-score	−1.68±1.05	−1.53±1.17	−0.965
Z-score	0.22±0.82	0.40±0.92	−1.452

Tested by independent *t*-test. Values are Mean±standard deviation.

Group 1: non-metabolic–syndrome group, Group 2: metabolic-syndrome group, BSA; body surface area, BMC; bone mineral content, BMD; bone mineral density

**Table 3:** Correlation between risk factors of metabolic syndrome and BMD-related factors

***Variable***	***BSA***	***BMC***	***BMD***	***T-score***	***Z-score***
WC	−0.242***	−0.113	−0.015	−0.024	0.011
SBP	0.132	0.112	0.072	0.071	0.101
DBP	0.186**	0.161*	0.111	0.109	0.129
TG	−0.061	0.019	0.047	0.048	0.057
HDL-C	0.007	−0.050	−0.071	−0.070	−0.074
FBS	0.104	0.077	0.049	0.045	0.063

All values but T and Z scores are t values from Pearson correlation test, BSA; body surface area, BMC; bone mineral content, BMD; bone mineral density, WC; waist circumference, SBP; systolic blood pressure, DBP; diastolic blood pressure, TG; triglyceride, HDL-C; high density lipoprotein cholesterol FBS; fasting blood sugar,

* *P*<.05

** *P*<.01

*** *P*<.001

The results of multiple regression analysis conducted to investigate the effect of risk factors of MetS on BMD are shown in Tables 4 and 5. When the T-score change was 24.0% and the F value was 0.600, none of the MetS risk factors showed a significant regression coefficient. In addition, when the Z-score change was 2.9% and the F-value was 0.733, a significant regression coefficient was not observed for any MetS risk factor.

**Table 4:** Multiple regression analysis of the effects of risk factors of metabolic-syndrome on T-score

***Variable***	***B***	***SE***	***β***	***t***	***P***
MetS	0.060	0.219	0.027	0.274	0.784
WC	−1.450	2.080	−0.052	−0.697	0.487
SBP	−0.005	0.010	−0.066	−0.462	0.645
DBP	0.018	0.014	0.165	1.249	0.213
TG	0.000	0.001	0.012	0.141	0.888
HDL-C	−0.009	0.010	−0.080	−0.918	0.360
FBS	0.002	0.005	0.031	0.420	0.675

B; unstandardized beta, SE; standard error, β; standardized beta, MetS; metabolic syndrome, WC; waist circumference, SBP; systolic blood pressure, DBP; diastolic blood pressure, TG; triglyceride, HDL-C; high density lipoprotein cholesterol, FBS; fasting blood sugar

**Table 5:** Multiple regression analysis of the effects of the risk factors of metabolic-syndrome on Z-score

***Variable***	***B***	***SE***	***β***	***t***	***P***
MetS	0.090	0.173	0.051	0.521	0.603
WC	−0.576	1.641	−0.026	−0.351	0.726
SBP	−0.002	0.008	−0.042	−0.295	0.768
DBP	0.013	0.011	0.148	1.118	0.265
TG	2.709	0.001	0.002	0.023	0.981
HDL-C	−0.008	0.008	−0.089	−1.033	0.303
FBS	0.002	0.004	0.044	0.600	0.549

B; unstandardized beta, SE; standard error, β; standardized beta, MetS; metabolic syndrome, WC; waist circumference, SBP; systolic blood pressure, DBP; diastolic blood pressure, TG; triglycerides, HDL-C; high density lipoprotein cholesterol, FBS; fasting blood sugar

## Discussion

This study aimed to investigate the relationship between BMD and risk factors of MetS in menopausal Korean women. The study results show that there was no statistically significant difference in BMD-related factors between the MetS and non-MetS groups. Pearson’s correlation analysis showed that there was a significant negative correlation between waist circumference and BSA of menopausal Korean women. Diastolic blood pressure was also positively correlated with BSA and BMC. However, multiple regression analysis did not reveal a significant relationship between BMD and risk factors of MetS These results suggest that the risk factors of MetS in menopausal Korean women have no significant effect on BMD.

The results of this study are similar to those of several previous studies (13, 20). There was no significant difference in lumbar or femoral neck densities between women with and without MetS in studies involving menopausal Iranian women (13). A study on menopausal Moroccan women revealed no difference in the incidence of vertebral fracture between women diagnosed with MetS and those who were not (20).

However, contrary to the present study, several studies have reported that MetS in postmenopausal women reduces BMD (4, 9). In addition, because BMD reduction has similar pathogenesis with arteriosclerosis, cardiovascular disease risk factors in menopausal women are associated with osteoporosis (30–31). In addition, the BMD of menopausal women with MetS was higher (32). This may have been because post-menopausal estrogen deficiency accompanies abdominal obesity and weight gain, resulting in an increase in the physical load on the bone and mitigating any decrease in BMD due to MetS (33).

Potential causes of such differences in outcomes among the studies include either the characteristics of the study subject groups, or genetic factors. A comparison of studies between those reporting relevant effects versus no relevance for MetS on BMD indicates that the studies involved different races or nationalities (4, 17, 32). For example, some works involved menopausal Turkish women (17, 23), and post-menopausal Tibetan women (4). Some studies have reported differences in bone health and bone density-related disease among races and countries (34), which have been confirmed by studies presenting the prevalence of osteoporosis in menopausal women in each country (35–36). For example, the prevalence of osteoporosis in the hip joint and lumbar spine of menopausal Moroccan women was 6.7% and 37.9%, respectively (35), and that of menopausal Saudi Arabian women was 44.1% and 46.7%, respectively (36).

This study had some limitations. First, since this study used a cross-sectional design, the effect of MetS on BMD in menopausal women over time could not be identified, and no clear causal relationship between the two variables could be determined. Second, this study involved only menopausal women living in Seoul, Korea. Therefore, it is difficult to generalize the findings to all menopausal women. Third, because the ages of the study subjects were limited to those in their 60s, results for older post-menopausal women were not analyzed. Fourth, bone–metabolism-related factors such as vitamin D, blood levels of calcium and osteocalcin, inflammatory factors, physical activity, exercise, eating behavior, smoking, alcohol consumption, and exposure to sunlight, all of which may affect BMD of menopausal women, were not investigated or measured in this study, which may confound the interpretation of the results of this study. Lastly, because BMD was measured only in the lumbar spine, the relationship between MetS and BMD at the femur in menopausal women was not confirmed.

## Conclusion

The risk factors of MetS in menopausal Korean women have no significant effect on BMD. Therefore, the role of risk factors of MetS in predicting BMD changes in menopausal Korean women seems to be limited. In follow-up studies, it is necessary to increase the size of the group and investigate other research variables

## Ethical considerations

Ethical issues (Including plagiarism, informed consent, misconduct, data fabrication and/or falsification, double publication and/or submission, redundancy, etc.) have been completely observed by the authors.
